# Enhancing the acceptance of smart sensing in psychotherapy patients: findings from a randomized controlled trial

**DOI:** 10.3389/fdgth.2024.1335776

**Published:** 2024-04-18

**Authors:** Fabian Rottstädt, Eduard Becker, Gabriele Wilz, Ilona Croy, Harald Baumeister, Yannik Terhorst

**Affiliations:** ^1^ Department of Clinical Psychology, Friedrich Schiller University of Jena, Jena, Germany; ^2^ DZPG (German Center for Mental Health), Partner Site Halle-Jena-Magdeburg, Jena, Germany; ^3^ Department of Clinical-Psychological Intervention, Friedrich Schiller University of Jena, Jena, Germany; ^4^ Department of Clinical Psychology and Psychotherapy, University Ulm, Ulm, Germany; ^5^ DZPG (German Center for Mental Health), Partner Site Mannheim-Ulm-Heidelberg, Ulm, Germany; ^6^ Department of Psychological Methods and Assessment, Ludwigs-Maximilian University Munich, Munich, Germany; ^7^ DZPG (German Center for Mental Health), Partner Site München, Munich, Germany

**Keywords:** smart sensing, psychotherapy, acceptance, digital health, implementation, unified theory of acceptance and use of technology

## Abstract

**Objective:**

Smart sensing has the potential to make psychotherapeutic treatments more effective. It involves the passive analysis and collection of data generated by digital devices. However, acceptance of smart sensing among psychotherapy patients remains unclear. Based on the unified theory of acceptance and use of technology (UTAUT), this study investigated (1) the acceptance toward smart sensing in a sample of psychotherapy patients (2) the effectiveness of an acceptance facilitating intervention (AFI) and (3) the determinants of acceptance.

**Methods:**

Patients (*N* = 116) were randomly assigned to a control group (CG) or intervention group (IG). The IG received a video AFI on smart sensing, and the CG a control video. An online questionnaire was used to assess acceptance of smart sensing, performance expectancy, effort expectancy, facilitating conditions and social influence. The intervention effects of the AFI on acceptance were investigated. The determinants of acceptance were analyzed with structural equation modeling (SEM).

**Results:**

The IG showed a moderate level of acceptance (*M* = 3.16, SD = 0.97), while the CG showed a low level (*M* = 2.76, SD = 1.0). The increase in acceptance showed a moderate effect in the intervention group (*p* < .05, *d* = 0.4). For the IG, performance expectancy (*M* = 3.92, SD = 0.7), effort expectancy (*M* = 3.90, SD = 0.98) as well as facilitating conditions (*M* = 3.91, SD = 0.93) achieved high levels. Performance expectancy (*γ* = 0.63, *p* < .001) and effort expectancy (*γ* = 0.36, *p* < .001) were identified as the core determinants of acceptance explaining 71.1% of its variance. The fit indices supported the model's validity (CFI = .95, TLI = .93, RMSEA = .08).

**Discussion:**

The low acceptance in the CG suggests that enhancing the acceptance should be considered, potentially increasing the use and adherence to the technology. The current AFI was effective in doing so and is thus a promising approach. The IG also showed significantly higher performance expectancy and social influence and, in general, a strong expression of the UTAUT factors. The results support the applicability of the UTAUT in the context of smart sensing in a clinical sample, as the included predictors were able to explain a great amount of the variance of acceptance.

## 1 Introduction

Digital technologies have the potential to significantly transform psychotherapeutic treatment and care (1–3). The hope is that they can help to bridge healthcare gaps and make treatments more effective and efficient (4–6). Smart sensing is one of those technologies that may contribute to improvements in psychotherapy. It primarily involves the passive analysis and collection of data generated by digital devices, such as smartphones or smart wearables (7, 8). Such data may encompass measurements like step counts, sleep duration, or smartphone usage. In the future, smart sensing could even capture more complex biophysiological data (9). This technology vastly extends the information available to psychotherapists during the treatment process. Smart sensing offers the distinct advantage that fine-grained data (e.g., continuous assessment of activity) can be collected unobtrusively without burden on patients. Furthermore, the technology enables more objective data collection in the natural life context of patients leading to high ecological validity and the elimination of common biases such as recall biases or social desirability, which have long posed challenges in psychotherapy (10, 11). The gathered information can be integrated at every stage of the psychotherapeutic process: diagnosis and problem analysis, treatment planning, implementation of interventions, monitoring, and the evaluation of the treatment process (3, 12).

Data collected through smart sensing has already been utilized in various domains of health research (13–15), such as measuring physical activity or sending activity-promoting app notifications in cases of extended sitting (16), or monitoring chronic conditions like Parkinson's disease (17). In the context of mental health conditions, smart sensing has also been employed (7, 8). From cross-sectional observation studies, there is evidence that mental symptoms are associated with smartphone or wearable data (18–20), which might enable predictions of mental disorders by this data in the future. There is also evidence for phenotyping and diagnosing diseases such as psychosis (21) and bipolar disorder (22) or for mood prediction (23, 24).

However, various steps need to be taken before integrating smart sensing technology into standard clinical care. One is to gauge its acceptability and discern the factors linked with its adoption. The Unified Theory of Acceptance and Use of Technology (UTAUT) (25) offers a model for investigating the acceptance of technology and its influencing factors. It is a well-established framework for understanding the adoption and acceptance of digital health applications (26, 27), and has already been applied to diverse contexts (27–29). The theory identifies performance expectancy, which relates to the perceived personal benefits of using the technology, effort expectancy, denoting the anticipated ease of use, social influence, representing the belief that others find the technology valuable, and facilitating conditions, encompassing the expected support and availability of practical resources, as the fundamental determinants of acceptance (25, 26). A first study also applied the UTAUT model in the context of smart sensing (30). While the study supports the general applicability of the UTAUT model for smart sensing, it was conducted in the general population and currently no evidence of the model is available in patients in psychotherapy. Hence, it is of importance to investigate the generalizability of the UTAUT model in a clinical sample.

In addition to understanding the determinates of acceptance, it is essential to explore opportunities for enhancing the acceptance to ensure the successful implementation of smart sensing. It has been proven before that Acceptance Facilitating Interventions (AFIs) can be effective in enhancing the acceptance of internet-based or blended psychotherapy (31–35). AFIs typically align with an acceptance model such as UTAUT (26) or other models [e.g., the Health Action Process Approach (36)]. To directly target the presumed determinants of acceptance, a UTAUT-based AFI should emphasize performance expectancy by pointing out the personal benefits, effort expectancy by demonstrating the technology in action, social influence by providing expert or user experiences, and facilitating conditions by addressing concerns regarding practical resources or the availability of technical assistance.

The present study aimed to (1) assess the acceptance toward smart sensing in a sample of psychotherapy patients (2) investigate the effectiveness of a UTAUT-based AFI in enhancing the acceptance of smart sensing and (3) investigate the determinants of acceptance. The AFI was presented to the intervention group (IG) in the form of an information video on smart sensing, while the control group (CG) was shown a control video that contained information about depression and anxiety (active control condition). Two hypotheses were investigated: (a) Patients who watched the AFI-video show a higher acceptance of smart sensing. (b) The UTAUT model applies to psychotherapy patients, which means that the covariance matrix implicated by the UTAUT does not differ significantly from the observed covariance matrix. In addition, we conducted exploratory analyses to assess the effect of the AFI on relevant subgroups and to investigate the association between psychological distress and the acceptance of smart sensing.

## 2 Materials and methods

### 2.1 Sample and study design

We report on a randomized controlled trial focusing on the cross-sectional comparison of two groups. The study was conducted in April and May, 2023. In this online intervention study patients were randomly assigned to either the control group (CG) or the intervention group (IG). A simple, unrestricted randomization was used, which was carried out by an automated and validated tool by the survey software (LimeSurvey Community Edition Version 6.2.9). The algorithm initially led to different group sizes (IG: 80, CG: 69). Assignment to CG or IG was obscured for participants. Patients in the IG received an informational video (AFI) on the topic of Smart sensing. Patients in the CG, on the other hand, received a video on the topic of depression and anxiety instead. Further details about the videos are provided in Section 2.5.1.

Patients were invited to participate via email. In order to be included in the study, patients either had to be undergoing psychotherapeutic treatment at the psychotherapeutic outpatient clinic of Friedrich-Schiller-University Jena, be on the waiting list for such treatment or have completed their psychotherapy within the last 2 years. Participants also had to have sufficient knowledge of the German language, and be over 18 years of age. Participants did not receive any reimbursements for participation.

To determine the required sample size, an *a priori* power analysis was conducted. Previous AFI interventions on acceptance ranged from no significant effects to significant effects with large effect sizes (31–34). Based on these study results, the effect size was assumed to be *d* = 0.40. With a power of 80%, a one-sided *t*-test and a significance level of 5%, a sample size of 78 participants per group was required.

**Figure 1:**
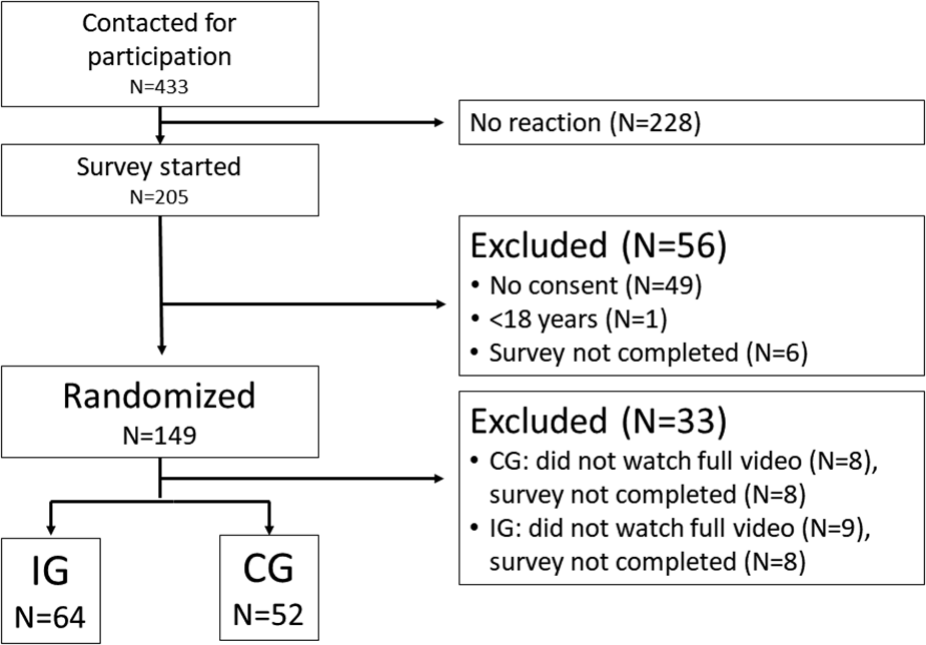
Study flow.

A total of 433 patients were contacted. Out of the contacted patients, 205 clicked on the study link. The final analysis included data from *N* = 116 patients, Figure 1 depicts the study flow.

Patient ages ranged from 18 to 87 years (*M* = 38.86, SD = 15.83). Of the patients, 63.8% were female (*n* = 74), 33.6% were male (*n* = 39), and 2.6% identified as gender-diverse (*n* = 3). Additionally, 39.7% had a university degree (*n* = 46). There were *n* = 64 patients in the intervention group and *n* = 52 in the control group. Table 1 presents sociodemographic data separately for both groups and Table 2 shows that both groups reported the same amount of psychological distress.

**Table 1:** Characteristics of IG and CG.

	Intervention group		Control group	
	*n* (*N* = 64)	%/M (SD)	*n* (*N* = 52)	%/M (SD)
Age in years		38.17 (15.06)		39.71 (15.87)
Sex				
Male	18	28.1	21	40.4
Female	44	68.8	30	57.7
Diverse	2	3.1	1	1.9
Country of origin				
Germany	59	92.2	52	100
Other	5	7.8	0	0
Education				
University degree	28	43.8	18	34.6
University entrance qualification	20	31.3	19	36.5
Intermediate secondary school	11	17.2	10	19.2
Other	5	7.8	5	9.6
Employment status				
Employed full-time	21	32.8	16	30.8
Education/study	19	29.7	15	28.8
Employed part-time	13	20.3	10	19.2
Retired	4	6.3	7	13.5
Other	7	10.9	4	7.7

M(SD), mean (standard deviation).

**Table 2:** Psychological distress of IG and CG.

		Intervention group *N* = 64				Control group *N* = 52			Comparison	
	M (SD)	Median (IQR)	Mean rank	Sum rank	M (SD)	Median (IQR)	Mean rank	Sum rank	*Z*	*p*
Depression	6.17 (5.17)	4.5 (7.0)	55.4	3,545.5	7.54 (5.88)	6.5 (8.0)	62.3	3,240.5	−1.11	.269
Anxiety	4.48 (3.41)	3.0 (4.0)	57.4	3,674.5	4.48 (3.03)	5.0 (5.0)	59.8	3,111.5	−0.39	.689
Stress	7.56 (4.01)	7.5 (5.0)	56.5	3,613.5	8.34 (4.81)	8.0 (8.0)	61.0	3,172.5	−0.73	.467
SUM	18.22 (10.38)	15.5 (13.75)	55.7	3,565.0	20.37 (12.21)	19.0 (18.0)	61.9	3,221.0	−0.99	.320

For comparison of both groups Mann-Whitney-*U*-test was used. M(SD), mean (standard deviation), Mean Rank, mean rank according to Mann-Whitney-*U*-test. Sum Rank, sum rank according to Mann-Whitney-*U*-test.

### 2.3 Ethics statement

The study followed the Declaration of Helsinki for medical research involving human subjects and was approved by the Ethics Committee of the Friedrich-Schiller University Jena (Reg.-Nr.: FSV 22/103). All participants provided written informed consent.

### 2.4 Questionnaires

#### 2.4.1 Acceptance

The assessment of acceptance and its determinants was grounded in the Unified Theory of Acceptance and Use of Technology (UTAUT) model (25). To gauge the efficacy of the intervention and the impact of these determinants, we utilized a slightly modified questionnaire, similar to those employed and validated in prior research endeavors aimed at measuring the acceptance of internet-based interventions (30, 31, 33, 35). The questionnaire comprises 5 scales (acceptance, performance expectancy, effort expectancy, social influence, facilitating conditions) and a total of 14 items. All items were rated on a 5-point Likert scale, ranging from 1 = “strongly disagree” to 5 = “strongly agree.” Detailed information on the questionnaire's scales and items, along with reliability statistics for this study, are presented in the supplement (Supplementary Tables S1 and S3).

#### 2.4.2 Psychological distress

Psychological distress was assessed using the German version of the Depression-Anxiety-Stress Scale (DASS) (37). The questionnaire comprises 21 items and gauges psychological distress across the domains of depression, stress, and anxiety, each consisting of seven items. Responses were captured on a 4-point Likert scale ranging from 0 = “Did not apply to me at all” to 3 = “Applied to me very much, or most of the time.” The DASS was selected due to its economy, ability to provide nuanced insights into various symptom domains of psychological distress, and established reliability and validity. Convergent validity was demonstrated through the correlation between the Beck Anxiety Inventory (38) and the DASS anxiety scale (*r* = .76), as well as the correlation between the Beck Depression Inventory (39) and the DASS depression scale (*r* = .68).

### 2.5 Material

#### 2.5.1 Intervention video

The AFI video had a duration of approximately 9 min. The video aimed to address the acceptance predictors outlined in the UTAUT model, with a particular focus on performance expectancy. The video was tailored to the target audience of psychotherapy patients, implemented a narrative style, and was based on a whiteboard design to achieve load reduction (40–42). Content within the video included an explanation of what smart sensing entails, how it can be utilized, information regarding data collection, data privacy considerations, and the benefits of smart sensing. The potential applications of smart sensing were further elucidated through a fictional patient scenario, highlighting the advantages of smart sensing. In this scenario, a therapist and a patient jointly recognize a positive correlation between physical activity and the patient's satisfaction. Subsequently, an objective is formulated within the therapy to increase physical activity. Furthermore, the video emphasized the use of smart sensing to monitor treatment progress and goal achievement during therapy, devising additional treatment objectives, and underlining the potential utility of smart sensing even beyond the course of therapy. Towards the end of the video, an expert statement from a psychotherapist was presented, expounding upon further advantages of Smart sensing and recounting personal positive experiences with it. A script outlining the video's content is provided in the appendix.

#### 2.5.2 Control video

The control video for the control group was thought as an active control condition. It had a duration of approximately 8.5 min. This video consisted of two psychoeducational segments, one addressing depression and the other focusing on generalized anxiety disorders. Both videos were created by the German Federal Ministry of Health and are publicly available (43, 44). These segments were amalgamated into a single video to ensure a comparable duration to that of the AFI Video. The aim of this video was to capture the participants’ attention due to the perceived importance of the subject matter without influencing their acceptance of smart sensing. Notably, smart sensing was not discussed in this video.

### 2.6 Implementation and Procedure

Patients initially completed the questionnaire pertaining to their sociodemographic characteristics. Subsequently, patients were presented with either the AFI video or control video. To ensure that the video was viewed, patients were required to wait for a minimum of 4 min before they could click on the “continue” button. Additionally, they were asked to confirm whether they had watched the video in its entirety. Following this, patients received information on smart sensing to ensure that even the CG had a basic understanding of the topic. The following information was provided: “Smart sensing involves the continuous passive collection of digital markers while using smart devices such as smartphones or smartwatches. Digital markers include parameters such as the range of motion, step count, and sleep patterns. Smart sensing, for instance, records your step count via a smartphone or your sleep patterns through a smartwatch.” Following the information session, patients completed the UTAUT questionnaire and the DASS. At the end of the study, participants were also given the option to receive automated feedback on the results of the DASS questionnaire. Furthermore, patients had the opportunity to provide their email address if they were interested in participating in further studies involving smart sensing.

### 2.7 Statistical analyses

The data was analyzed using IBM SPSS Statistics 29.00 and IBM SPSS Amos 29. To compare level of acceptance between IG and CG, the Mann-Whitney *U* test was employed due to deviations from the assumption of normal distribution. To examine the influence of the surveyed acceptance predictors (performance expectancy, effort expectancy, social influence, facilitating conditions), a confirmatory structural equation model was applied to the whole sample, whereby the CG and IG were combined. The structural equation model was developed based on UTAUT and validated by Philippi et al. (27). The influence of the acceptance predictors on acceptance was tested using standardized regression weights and their significance. Model fit was evaluated using the Comparative Fit Index (CFI), the Tucker Lewis Index (TLI) and the Root Mean Square Error of Approximation (RMSEA). RMSEA as a non-centrality parameter was used to assess the goodness of fit due to the tendency of the *χ*^2^-test to reject the misspecified models too harshly (45–47). Following well-established guidelines we defined a good model fit as CFI and TLI >.90, RMSEA <.08 (48).

Additionally, exploratory *t*-tests were conducted. The effects of the intervention on various subgroups were also examined to analyze in which groups the intervention is particularly meaningful and to explore potential mechanisms of the intervention. Exploratory analyses were performed for the following subgroups: male vs. female, young vs. old (with the median age as the cutoff point) as well as lower vs. higher educational levels (higher degrees beyond secondary school were considered as higher education). To investigate the association between acceptance and psychological distress, Pearson correlation coefficient was computed.

## 3 Results

### 3.1 Group Comparison of acceptance and its predictors

On a 5-point Likert scale, the IG showed a moderate level of acceptance (*M* = 3.16), while the CG showed a low level (*M* = 2.76). Table 3 presents the comparison of groups in terms of acceptance and acceptance predictors. The IG showed significantly higher acceptance than CG (*U* = 1,299.0, *Z* = −2.033, *p* = .042). The effect size was *d* = 0.40 (95% CI: 0.03–0.77), corresponding to a moderate effect. Hence, hypothesis (a) was confirmed. Furthermore, patients in the IG had significantly higher performance expectancy toward smart sensing (*U* = 2,404.5, *Z* = −3.575, *p* < .001), with an effect size of *d* = 0.74 (95% CI: 0.36–1.11), as well as significantly higher scores on social influence (*U* = 1,056.0, *Z* = 3.460, *p* < .001), with an effect size of *d* = 0.63 (95% CI: 0.25–1.00).

**Table 3:** Acceptance and predictors of acceptance for smart sensing.

	Intervention group *N* = 64			Control group *N* = 52			Comparison		
	M (SD)	Mean rank	Sum rank	M (SD)	Mean rank	Sum rank	*Z*	*p*	*d*
Acceptance	3.16 (0.97)	64.2	4,109.0	2.76 (1.00)	51.48	2,677.0	−2.03	**<**.05*	**0**.**4**
Performance expectancy	3.92 (0.70)	68.5	4,381.5	3.31 (0.97)	46.2	2,404.5	−3.60	**<**.001***	**0**.**74**
Effort expectancy	3.90 (0.98)	63.6	4,072.0	3.60 (1.02)	52.2	2,714.0	−1.84	.065	
Social influence	3.34 (0.83)	68.0	4,352.0	2.83 (0.79)	46.8	2,434.0	−3.50	**<**.001***	**0**.**63**
Facilitating conditions	3.91 (0.93)	59.6	3,812.5	3.82 (1.00)	57.2	2,973.5	−0.39	.698	

For comparison of both groups Mann-Whitney-*U*-test was used. M(SD), mean (standard deviation), Mean Rank, mean rank according to Mann-Whitney-*U*-test. Sum Rank, sum rank according to Mann-Whitney-*U*-test.

Significant *p*-values are written in bold.

*<.05, ***<.001.

### 3.2 Model for prediction of acceptance of smart sensing

**Figure 2:**
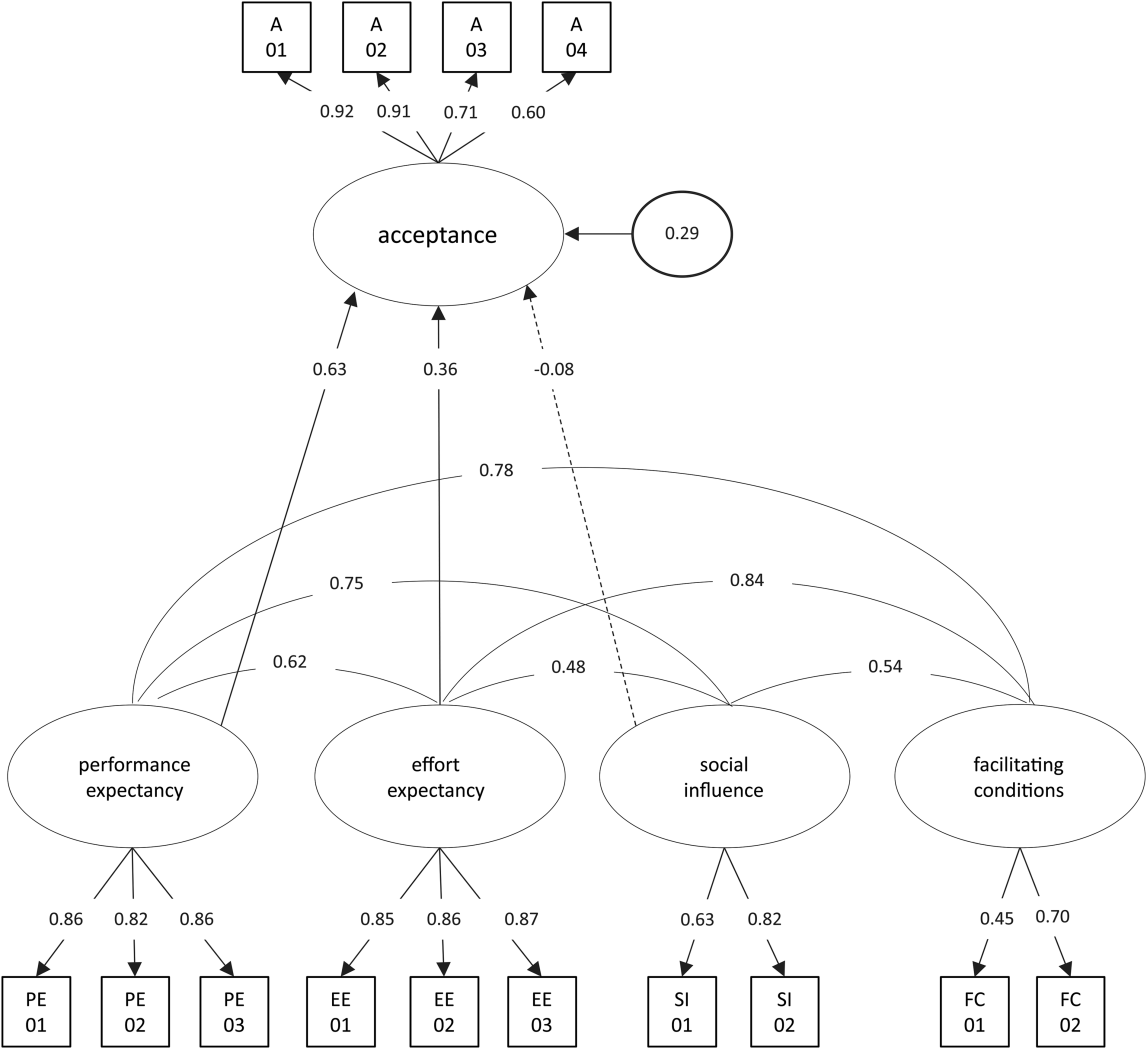
Structural equation model for the acceptance toward smart sensing. Latent variables are represented in ellipses: A, acceptance; PE, performance expectancy; EE, effort expectancy; FC, facilitating conditions; SI, social influence. The dashed line indicates a nonsignificant path. Observed items are indicated as rectangles. Path loadings are represented as single-headed arrows. All exogenous latent variables were allowed to correlate. For improved readability, all latent correlations and residual variances of manifest items were omitted.

Performance expectancy (*γ* = 0.63, *p* < .001) and effort expectancy (*γ* = 0.36, *p* < .001) were identified as predictors of acceptance in the structural equation model. Together, the two determinants explained 71.1% of the variance of the latent acceptance factor. Social influence did not achieve statistical significance as a predictor of acceptance (*γ* = −0.08, *p* = .551). The fit indices supported the model's validity (CFI = .95, TLI = .93, RMSEA = .08). Hence, hypothesis (b) was confirmed. Figure 2 depicts the structural equation model with estimated parameters. The full parameter list of the measurement model and correlations between the acceptance predictors and acceptance of smart sensing are presented in the supplement (Supplementary Tables S2 and S3).

### 3.3 Exploratory analyses

#### 3.3.1 Subgroup analyses

Subgroup analyses revealed meaningful interaction effects regarding gender, age, and education level. A significant intervention effect on acceptance was found in the subgroup of women (*t* = 3.54, *p* < .001) with an effect size of *d* = 0.84 (95% CI: 0.35–1.32). Conversely, there was no significant effect in men (*t* = −0.59, *p* = .561). Furthermore, a significant intervention effect was observed in the older patient group (*t* = 2.37, *p* = .021) with an effect size of *d* = 0.62 (95% CI: 0.09–1.14), whereas there was no significant effect in the younger patient group (*t* = 0.55, *p* = .586). Similarly, a significant intervention effect was observed for patients with lower educational levels [*t* = 2.92, *p* = .007, *d* = 1.15 (95% CI: 0.30–1.97)], but not for patients with higher education (*t*_ _= 1.56, *p* = .114). Table 4 includes the means and standard deviations of acceptance for each subgroup, along with the *p*-values of the intervention effects.

**Table 4:** Subgroup analyses of acceptance.

	Intervention group *N *= 64			Control group *N *= 52			Comparison		
	*N*	*M*	SD	*N*	*M*	SD	df	*t*	*p*
Sex									
Male	18	3.06	1.06	21	3.25	1.00	37	−0.59	.561
Female	44	3.22	0.96	30	2.45	0.86	72	3.54	<.001
Age in years									
<34	34	3.16	0.86	26	3.03	0.98	48	0.55	.586
≧34	30	3.15	1.09	26	2.51	0.96	54	2.37	.021
Education									
Low	16	3.52	0.85	15	2.58	0.79	39	2.92	.007
High	48	3.16	0.93	37	2.82	1.06	83	1.56	.114

M(SD), mean (standard deviation), For the separation between older and younger patients, the median age (34) of the entire sample was used high educational status = any person holding a university entrance qualification or higher.

### 3.3.2 Psychological distress and acceptance

There were no significant associations in the overall sample between acceptance and depression [*r* = .02 (95% CI: −.17 to −.20), *p* = .867], acceptance and anxiety [*r* = .01 (95% CI: −.17 to −.20), *p* = .893], acceptance and stress [*r* = .06 (95% CI: −.13 to −.24), *p* = .560], or between acceptance and overall psychological distress [*r* = .03 (95% CI: −.15 to −.21), *p* = .728].

## 4 Discussion

### 4.1 Effect of AFI

Acceptance is a fundamental precondition for the dissemination, uptake, and clinical impact of smart sensing. The mean acceptance of the CG suggests that the baseline acceptance for smart sensing in psychotherapy patients is at a low to moderate level. We hence conclude that enhancing the acceptance should be considered, potentially increasing the use and adherence to the technology. The present UTAUT-based AFI was able to significantly increase the acceptance of smart sensing in psychotherapy patients and increased performance expectancy and social influence as well. The effect size of the intervention on acceptance (*d* = 0.40) fell within moderate range. Considering determinants of acceptance according to the UTAUT model, performance and effort expectancy achieved high levels with means close to 4 on a 5-point Likert scale in the IG (compare Table 4). Social influence was increased by the AFI reaching a moderate to high level. Additionally, the overall sample showed high levels of facilitating conditions.

To the best of our knowledge, this is the first study to explore the use of an AFI for smart sensing in psychotherapy patients. Consequently, a direct comparison with study outcomes in similar clinical populations is not feasible. Compared to the general population (30), psychotherapy patients exhibited similar levels of acceptance for smart sensing in the control group, while this study revealed a greater intervention effect by the AFI. We identified the following reasons for the greater intervention effect: (1) The AFI in this study was tailored to the group of psychotherapy patients and specifically targeted their needs, whereas the AFI in the comparative study did not address specific needs but introduced the technology and possible applications in a more general manner. (2) The AFI in this study was three times longer than in the comparative study, which allowed us to provide more in-depth information about smart sensing. (3) Performance expectancy was the most important predictor of acceptance for smart sensing in both this patient population and the general population (30). However, it is questionable whether a population with good mental health expects meaningful benefits from smart sensing, while a population of psychotherapy patients might have a clearer connection to the benefits of the technology. Lastly, we optimized the intervention by applying state of the art instructional design principles to split the cognitive load across auditory and visual channels in the white-board video and implemented a narrative explanation style in the AFI (40–42).

For clinical practice our results support the implementation of scalable AFI in an online video format to increase the acceptance of smart sensing in patients. Given the time- and location independent nature of such AFI, they may become a feasible and effective way to implement smart sensing at various stages before (e.g., installation and symptom tracking before treatment for a data informed decision and recommendation of psychotherapeutic modules), during (e.g., to monitor treatment progress via smart sensing), and after psychotherapy [e.g., using smart sensing to recognize re-establishing dysfunctional behavior patterns and initiate just-in-time interventions (3)]. That said, the therapeutic relationship between the patient and therapist (49) represents one of the most crucial therapeutic factors in psychotherapy. Hence, it is reasonable to assume that a recommendation for the use of smart sensing by the treating psychotherapist would significantly enhance the acceptance and could outperform the effects of digital AFI. The evaluation of expert-delivered face-to-face AFIs or stepped-information processes combining digital and face-to-face AFIs would be a very valuable addition to this study.

### 4.2 Influence of acceptance predictors

We confirmed our hypothesis that the UTAUT holds in the context of smart sensing in a clinical sample as the included predictors explained a great amount of the variance of acceptance. This finding aligns with previous findings (30). The results also emphasize that the most critical factor for the acceptance of smart sensing is the expected personal benefit to the patient. Also consistent with prior research, the second most influential predictor was effort expectancy, albeit with a significantly greater impact on acceptance than could be expected based on previous findings (27, 30). One possible reason for this could be that a common symptom in mental disorders, particularly in cases of depressive symptoms, is aversion or a lack of motivation (50). Therefore, any additionally perceived effort is likely to have a more negative impact on acceptance in psychotherapy patients compared to other populations. Thus, the perceived minimal effort appears to be of importance for the acceptance of smart sensing in this context. While social influence showed a significant positive correlation with acceptance, social influence did not remain a predictor of acceptance in the structural equation model. This could be attributed to its contribution to the explained variance in acceptance, which was already accounted for by performance expectancy and effort expectancy, both of which were also significantly correlated with social influence.

Hence, we strongly recommend to focus on the performance expectancy when aiming to successfully implement smart sensing in clinical practice as the influence is almost twice as strong than effort expectancy. For instance, this could be done by highlighting the benefits of smart sensing and how the psychotherapeutic process can benefit from it (e.g., trajectory modeling, early-warning systems). Besides, the already outlined potential to increase the feasibility of smart sensing in clinical practice, AFI may also hold the potential to increase the adherence to smart sensing sample protocols in research to counteract missingness and increase data quality (18, 19, 51).

### 4.3 Exploratory analyses

The Subgroup analyses are of particular interest when it comes to the question for which subgroups the AFI had the largest impact. However, these analyses must be interpreted in light of the fact that the group sizes were neither large nor balanced with respect to key individual variables. Therefore, they can only provide a hint for future research questions of interest. The results indicate that the AFI particularly enhanced acceptance among females, older patients, and patients with lower education. This appears to be due to lower baseline acceptance of smart sensing in these groups compared to their respective counterparts. This suggests that those are the groups that should especially be provided with an AFI when smart sensing is recommended. Future research is necessary to replicate those findings and might test if AFIs that target specific needs or concerns of those groups could further enhance the acceptance of smart sensing.

Neither stress, anxiety, depression, nor overall psychological distress exhibited a significant association with acceptance. This finding contrasts with other studies reporting a positive relationship between symptom severity and the acceptance of modern technologies in treatment (31, 35, 52). However, it should be noted that the studies by Lin et al. (35) and Baumeister et al. (31) examined different patient groups (pain patients and diabetes patients) and focused on Internet- and mobile-based interventions.

### 4.5 Limitations

It is important to address certain limitations when interpreting the results. (1) The present study was designed to investigate the acceptance of smart sensing in psychotherapy patients, but did not make a differentiation between mental diagnoses, which could have an impact on the acceptance. To infer to moderation effects on the symptomology level, we conducted exploratory correlation analyses between depression, anxiety, stress, and distress, which yielded non-significant findings. Besides, a clinical discussion for which patient groups the technology might be suited at all, future studies should explore the acceptance in more detail in specific patient groups. (2) Despite contacting every potentially available patient at the site twice, only 149 patients could be recruited for randomization. This means that the final recruitment target of 156 patients could not be reached. At this point, the recruitment capacity at the site was exhausted in terms of patients. Future studies should aim for a confirmatory study and could base their calculations on an effect size of *d* = 0.4. (3) The active control condition might also have had an effect on the acceptability towards mental health interventions in general, which might carry over to smart sensing. This would mean that baseline acceptance might be even lower, which could be investigated in future studies. (4) Future studies should follow-up with a closer investigation of the acceptance towards specific sensor modalities, such as screen usage, location, biophysiological data or language usage. For instance, Nicholas and colleagues (53) found differences in the acceptance towards health information (e.g., sleep, mood data), and personal data (e.g., communication logs, or location features). To which extent the acceptance might vary across sensors in psychotherapy patients is currently unknown. (5), the present sample showed an imbalance in gender and education leaning towards a female highly educated population. While this may reflect imbalances in prevalence rates for some disorders (e.g., increased prevalence of depression in women) and help seeking behavior to some extent, it also limits the generalizability of the present findings highlighting the need for replication studies. The slight imbalances between the IG and CG concerning gender and education were due to the randomization process but did not influence the overall effect of the AFI on acceptance (please compare Supplementary Material S5). Lastly, like many previous studies, this research primarily assessed acceptance and attitudes towards new technology by predicting behavioral intentions. While behavioral intentions are widely recognized as a proximal indicator of actual behavior, a gap often exists between intention and behavior (54). Therefore, future research should take into account the volitional aspect and incorporate actual smart sensing use, such as uptake rates, as an outcome measure (30, 36, 55).

## 5 Conclusion

In summary, our study provides evidence that acceptance of smart sensing among psychotherapy patients can be significantly increased by an AFI based on a time- and location independent video format. The low to moderate baseline acceptance level in the CG simultaneously emphasizes the importance of such interventions to potentially ensure technology usage and compliance.

Our IG exhibited high levels of performance expectancy, effort expectancy, and facilitating conditions after exposure to the AFI. This outcome is particularly promising from a scalability perspective, as such videos offer a versatile means of dissemination through various communication channels, including waiting rooms, the Internet, or television. This widespread distribution can significantly contribute to the adoption of these innovative digital health applications.

The study demonstrates that the UTAUT model is applicable within the context of smart sensing in a clinical sample. The findings highlight that the most critical factor for the acceptance of smart sensing is performance expectancy. Therefore, when recommending smart sensing to patients, the focus should be on their expected personal benefits. Exploratory findings suggest that this approach may be especially beneficial for increasing acceptance among females, older patients, and those with lower levels of education.

## Data Availability

Data requests should be directed to the corresponding author (FR). Data can be shared with researchers who provide a methodologically sound proposal, which is not already covered by other researchers. Data can only be shared for projects if the General Data Protection Regulation is met. Requestors may need to sign additional data access agreements. Support depends on available resources.
