# Supplementary material for: Enhancing the acceptance of smart sensing in psychotherapy patients: findings from a randomized controlled trial
Source: Front Digit Health. 2024 Apr 18;6:1335776. doi: 10.3389/fdgth.2024.1335776 (PMC11063245; doi:10.3389/fdgth.2024.1335776)
Supplement: Supplementary file 1 [file Table1.docx]

Supplementary material

**Supplement 1**

Table S1 Items for measuring acceptance and acceptance predictors based on the UTAUT model.

| Scale | Items | Reliability |
| --- | --- | --- |
| acceptance | 1. I could imagine using a smart sensing app. 2. If offered, I would use a smart sensing app regularly 3. I would recommend a smart sensing app to a friend 4. I would be willing to pay for a smart sensing app | .86 |
| performance expectancy | 1. Using a smart sensing app could have positive effects on my mental health. 2. Using a smart sensing app and the collected data could help my psychotherapist in diagnoses 3. Overall, a smart sensing app could help managing mental health issues. | .88 |
| effort expectancy | 1. Using a smart sensing app would be simple 2. Using a smart sensing app would be an easy task for me 3. A smart sensing app would be clear and easily comprehensible to me | .89 |
| social influence | 1. People close to me would recommend me to use a smart sensing app 2. My psychotherapist would recommend me to use a smart sensing app | .68 |
| facilitating conditions | 1. I have all necessary technical preconditions for using a smart sensing app 2. In case of technical problems with a smart sensing app I would receive technical support |  |

Reliability was reported using Cronbach's Alpha (α). The reliability regarding facilitating conditions was not provided because the items do not measure a consistent scale.

**Supplement 2: Drop out analysis**

To validate that the differences in dropout do not signficantly differ, we have ran a Chi-square test on the drop-out and completer rates yielding no significant differences at p < .05 (χ 2 χ^2^ = 0.14; p = .71). The chi-square statistic with Yates correction was also not significant at p < .05 (χ^2^ = 0.03; p = .86).

Table S2 Drop-out analysis

|  | | | |
| --- | --- | --- | --- |
|  | DropOut | Completer | Marginal Row Totals |
| IG | 17   (17.94)   [0.05] | 64   (63.06)   [0.01] | 81 |
| CG | 16   (15.06)   [0.06] | 52   (52.94)   [0.02] | 68 |
| Marginal Column Totals | 33 | 116 | 149    (Grand Total) |

**Supplement 3**

Table S3 Correlations between acceptance and its predictors

| Variable | 1 | 2 | 3 | 4 |
| --- | --- | --- | --- | --- |
| 1. acceptance |  |  |  |  |
| 1. performance expectancy | .75**  [.65, .82] |  |  |  |
| 1. effort expectancy | .65**  [.52, .74] | .55**  [.41, .67] |  |  |
| 1. social influence | .47**  [.31, .60] | .58**  [.45, .70] | .37**  [.20, .52] |  |
| 1. facilitating conditions | .49**  [.33, .61] | .53**  [.38, .65] | .58**  [.45, .69] | .31**  [.13, .46] |

Values in parentheses indicate the 95% confidence interval. ** = p < .001

**Supplement 4**

# Full model parameters & reliability

A full list of all parameters in the measurement model (latent variables, covariances, and variances) can be found below. Acceptance is abbreviated (BI), performance expectancy (PE), effort expectancy (EE), facilitating conditions (FC), social influence (SI).

The model is recursive.

Sample size = 116

## Variable Summary (Group number 1)

## Your model contains the following variables (Group number 1)

Observed, endogenous variables

UTAUT1_BI04

UTAUT1_BI03

UTAUT1_BI02

UTAUT1_BI01

UTAUT1_PE01

UTAUT1_PE02

UTAUT1_PE03

UTAUT1_EE01

UTAUT1_EE02

UTAUT1_EE03

UTAUT4_SI01

UTAUT4_SI02

UTAUT4_FC01

UTAUT4_FC02

Unobserved, endogenous variables

BI

Unobserved, exogenous variables

e1

e2

e3

e4

PE

e5

e6

e7

EE

e8

e9

e10

SI

e11

e12

FC

e13

e14

e15

## Variable counts (Group number 1)

| **Number of variables in your model:** | 34 |
| --- | --- |
| **Number of observed variables:** | 14 |
| **Number of unobserved variables:** | 20 |
| **Number of exogenous variables:** | 19 |
| **Number of endogenous variables:** | 15 |

## Parameter Summary (Group number 1)

|  | **Weights** | **Covariances** | **Variances** | **Means** | **Intercepts** | **Total** |
| --- | --- | --- | --- | --- | --- | --- |
| **Fixed** | 20 | 0 | 0 | 19 | 1 | 40 |
| **Labeled** | 0 | 0 | 0 | 0 | 0 | 0 |
| **Unlabeled** | 12 | 6 | 19 | 0 | 14 | 51 |
| **Total** | 32 | 6 | 19 | 19 | 15 | 91 |

## Models

## Default model (Default model)

## Notes for Model (Default model)

## Computation of degrees of freedom (Default model)

| **Number of distinct sample moments:** | 119 |
| --- | --- |
| **Number of distinct parameters to be estimated:** | 51 |
| **Degrees of freedom (119 - 51):** | 68 |

## Result (Default model)

Minimum was achieved

Chi-square = 116,866

Degrees of freedom = 68

Probability level = ,000

## Group number 1 (Group number 1 - Default model)

## Estimates (Group number 1 - Default model)

## Scalar Estimates (Group number 1 - Default model)

## Maximum Likelihood Estimates

## Regression Weights: (Group number 1 - Default model)

|  |  |  | **Estimate** | **S.E.** | **C.R.** | **P** | **Label** |
| --- | --- | --- | --- | --- | --- | --- | --- |
| BI | <--- | PE | ,461 | ,121 | 3,821 | *** |  |
| BI | <--- | EE | ,261 | ,074 | 3,510 | *** |  |
| BI | <--- | SI | -,083 | ,138 | -,597 | ,551 |  |
| UTAUT1_BI04 | <--- | BI | 1,000 |  |  |  |  |
| UTAUT1_BI03 | <--- | BI | 1,217 | ,196 | 6,214 | *** |  |
| UTAUT1_BI02 | <--- | BI | 1,694 | ,231 | 7,319 | *** |  |
| UTAUT1_BI01 | <--- | BI | 1,821 | ,247 | 7,363 | *** |  |
| UTAUT1_PE01 | <--- | PE | 1,000 |  |  |  |  |
| UTAUT1_PE02 | <--- | PE | ,819 | ,077 | 10,630 | *** |  |
| UTAUT1_PE03 | <--- | PE | ,932 | ,081 | 11,465 | *** |  |
| UTAUT1_EE01 | <--- | EE | 1,000 |  |  |  |  |
| UTAUT1_EE02 | <--- | EE | 1,120 | ,099 | 11,320 | *** |  |
| UTAUT1_EE03 | <--- | EE | 1,048 | ,091 | 11,545 | *** |  |
| UTAUT4_SI01 | <--- | SI | 1,000 |  |  |  |  |
| UTAUT4_SI02 | <--- | SI | 1,188 | ,223 | 5,326 | *** |  |
| UTAUT4_FC01 | <--- | FC | 1,000 |  |  |  |  |
| UTAUT4_FC02 | <--- | FC | 1,815 | ,432 | 4,200 | *** |  |

## Standardized Regression Weights: (Group number 1 - Default model)

|  |  |  | **Estimate** |
| --- | --- | --- | --- |
| BI | <--- | PE | ,635 |
| BI | <--- | EE | ,358 |
| BI | <--- | SI | -,080 |
| UTAUT1_BI04 | <--- | BI | ,604 |
| UTAUT1_BI03 | <--- | BI | ,706 |
| UTAUT1_BI02 | <--- | BI | ,906 |
| UTAUT1_BI01 | <--- | BI | ,917 |
| UTAUT1_PE01 | <--- | PE | ,859 |
| UTAUT1_PE02 | <--- | PE | ,816 |
| UTAUT1_PE03 | <--- | PE | ,858 |
| UTAUT1_EE01 | <--- | EE | ,854 |
| UTAUT1_EE02 | <--- | EE | ,858 |
| UTAUT1_EE03 | <--- | EE | ,870 |
| UTAUT4_SI01 | <--- | SI | ,628 |
| UTAUT4_SI02 | <--- | SI | ,817 |
| UTAUT4_FC01 | <--- | FC | ,450 |
| UTAUT4_FC02 | <--- | FC | ,704 |

## Intercepts: (Group number 1 - Default model)

|  |  |  | **Estimate** | **S.E.** | **C.R.** | **P** | **Label** |
| --- | --- | --- | --- | --- | --- | --- | --- |
| **UTAUT1_BI04** |  |  | 2,147 | ,101 | 21,220 | *** |  |
| **UTAUT1_BI03** |  |  | 3,190 | ,105 | 30,300 | *** |  |
| **UTAUT1_BI02** |  |  | 3,164 | ,114 | 27,711 | *** |  |
| **UTAUT1_BI01** |  |  | 3,414 | ,121 | 28,154 | *** |  |
| **UTAUT1_PE01** |  |  | 3,414 | ,098 | 34,832 | *** |  |
| **UTAUT1_PE02** |  |  | 3,914 | ,084 | 46,345 | *** |  |
| **UTAUT1_PE03** |  |  | 3,612 | ,091 | 39,501 | *** |  |
| **UTAUT1_EE01** |  |  | 3,655 | ,098 | 37,285 | *** |  |
| **UTAUT1_EE02** |  |  | 3,724 | ,109 | 34,093 | *** |  |
| **UTAUT1_EE03** |  |  | 3,888 | ,101 | 38,571 | *** |  |
| **UTAUT4_SI01** |  |  | 2,974 | ,094 | 31,500 | *** |  |
| **UTAUT4_SI02** |  |  | 3,241 | ,086 | 37,580 | *** |  |
| **UTAUT4_FC01** |  |  | 3,603 | ,101 | 35,720 | *** |  |
| **UTAUT4_FC02** |  |  | 4,129 | ,117 | 35,250 | *** |  |

## Covariances: (Group number 1 - Default model)

|  |  |  | **Estimate** | **S.E.** | **C.R.** | **P** | **Label** |
| --- | --- | --- | --- | --- | --- | --- | --- |
| PE | <--> | EE | ,505 | ,104 | 4,876 | *** |  |
| PE | <--> | SI | ,429 | ,101 | 4,248 | *** |  |
| FC | <--> | PE | ,343 | ,094 | 3,630 | *** |  |
| EE | <--> | SI | ,276 | ,082 | 3,356 | *** |  |
| FC | <--> | EE | ,366 | ,098 | 3,720 | *** |  |
| FC | <--> | SI | ,167 | ,062 | 2,681 | ,007 |  |

## Correlations: (Group number 1 - Default model)

|  |  |  | **Estimate** |
| --- | --- | --- | --- |
| PE | <--> | EE | ,624 |
| PE | <--> | SI | ,747 |
| FC | <--> | PE | ,780 |
| EE | <--> | SI | ,483 |
| FC | <--> | EE | ,837 |
| FC | <--> | SI | ,539 |

## Variances: (Group number 1 - Default model)

|  |  |  | **Estimate** | **S.E.** | **C.R.** | **P** | **Label** |
| --- | --- | --- | --- | --- | --- | --- | --- |
| **PE** |  |  | ,814 | ,146 | 5,591 | *** |  |
| **EE** |  |  | ,805 | ,146 | 5,531 | *** |  |
| **SI** |  |  | ,405 | ,125 | 3,246 | ,001 |  |
| **FC** |  |  | ,237 | ,107 | 2,221 | ,026 |  |
| **e15** |  |  | ,121 | ,039 | 3,099 | ,002 |  |
| **e1** |  |  | ,748 | ,103 | 7,256 | *** |  |
| **e2** |  |  | ,639 | ,091 | 7,011 | *** |  |
| **e3** |  |  | ,268 | ,057 | 4,688 | *** |  |
| **e4** |  |  | ,268 | ,062 | 4,295 | *** |  |
| **e5** |  |  | ,290 | ,054 | 5,370 | *** |  |
| **e6** |  |  | ,274 | ,045 | 6,023 | *** |  |
| **e7** |  |  | ,254 | ,047 | 5,387 | *** |  |
| **e8** |  |  | ,300 | ,056 | 5,401 | *** |  |
| **e9** |  |  | ,363 | ,068 | 5,319 | *** |  |
| **e10** |  |  | ,284 | ,056 | 5,046 | *** |  |
| **e11** |  |  | ,620 | ,103 | 6,007 | *** |  |
| **e12** |  |  | ,284 | ,097 | 2,942 | ,003 |  |
| **e13** |  |  | ,933 | ,134 | 6,980 | *** |  |
| **e14** |  |  | ,797 | ,202 | 3,951 | *** |  |

## Squared Multiple Correlations: (Group number 1 - Default model)

|  |  |  | **Estimate** |
| --- | --- | --- | --- |
| **BI** |  |  | ,717 |
| **UTAUT4_FC02** |  |  | ,495 |
| **UTAUT4_FC01** |  |  | ,203 |
| **UTAUT4_SI02** |  |  | ,668 |
| **UTAUT4_SI01** |  |  | ,395 |
| **UTAUT1_EE03** |  |  | ,757 |
| **UTAUT1_EE02** |  |  | ,736 |
| **UTAUT1_EE01** |  |  | ,729 |
| **UTAUT1_PE03** |  |  | ,736 |
| **UTAUT1_PE02** |  |  | ,666 |
| **UTAUT1_PE01** |  |  | ,737 |
| **UTAUT1_BI01** |  |  | ,842 |
| **UTAUT1_BI02** |  |  | ,821 |
| **UTAUT1_BI03** |  |  | ,498 |
| **UTAUT1_BI04** |  |  | ,365 |

## Matrices (Group number 1 - Default model)

## Total Effects (Group number 1 - Default model)

|  | **SI** | **EE** | **PE** | **FC** | **BI** |
| --- | --- | --- | --- | --- | --- |
| **BI** | -,083 | ,261 | ,461 | ,000 | ,000 |
| **UTAUT4_FC02** | ,000 | ,000 | ,000 | 1,815 | ,000 |
| **UTAUT4_FC01** | ,000 | ,000 | ,000 | 1,000 | ,000 |
| **UTAUT4_SI02** | 1,188 | ,000 | ,000 | ,000 | ,000 |
| **UTAUT4_SI01** | 1,000 | ,000 | ,000 | ,000 | ,000 |
| **UTAUT1_EE03** | ,000 | 1,048 | ,000 | ,000 | ,000 |
| **UTAUT1_EE02** | ,000 | 1,120 | ,000 | ,000 | ,000 |
| **UTAUT1_EE01** | ,000 | 1,000 | ,000 | ,000 | ,000 |
| **UTAUT1_PE03** | ,000 | ,000 | ,932 | ,000 | ,000 |
| **UTAUT1_PE02** | ,000 | ,000 | ,819 | ,000 | ,000 |
| **UTAUT1_PE01** | ,000 | ,000 | 1,000 | ,000 | ,000 |
| **UTAUT1_BI01** | -,150 | ,476 | ,839 | ,000 | 1,821 |
| **UTAUT1_BI02** | -,140 | ,443 | ,780 | ,000 | 1,694 |
| **UTAUT1_BI03** | -,100 | ,318 | ,560 | ,000 | 1,217 |
| **UTAUT1_BI04** | -,083 | ,261 | ,461 | ,000 | 1,000 |

## Standardized Total Effects (Group number 1 - Default model)

|  | **SI** | **EE** | **PE** | **FC** | **BI** |
| --- | --- | --- | --- | --- | --- |
| **BI** | -,080 | ,358 | ,635 | ,000 | ,000 |
| **UTAUT4_FC02** | ,000 | ,000 | ,000 | ,704 | ,000 |
| **UTAUT4_FC01** | ,000 | ,000 | ,000 | ,450 | ,000 |
| **UTAUT4_SI02** | ,817 | ,000 | ,000 | ,000 | ,000 |
| **UTAUT4_SI01** | ,628 | ,000 | ,000 | ,000 | ,000 |
| **UTAUT1_EE03** | ,000 | ,870 | ,000 | ,000 | ,000 |
| **UTAUT1_EE02** | ,000 | ,858 | ,000 | ,000 | ,000 |
| **UTAUT1_EE01** | ,000 | ,854 | ,000 | ,000 | ,000 |
| **UTAUT1_PE03** | ,000 | ,000 | ,858 | ,000 | ,000 |
| **UTAUT1_PE02** | ,000 | ,000 | ,816 | ,000 | ,000 |
| **UTAUT1_PE01** | ,000 | ,000 | ,859 | ,000 | ,000 |
| **UTAUT1_BI01** | -,074 | ,328 | ,582 | ,000 | ,917 |
| **UTAUT1_BI02** | -,073 | ,324 | ,575 | ,000 | ,906 |
| **UTAUT1_BI03** | -,057 | ,253 | ,448 | ,000 | ,706 |
| **UTAUT1_BI04** | -,048 | ,216 | ,383 | ,000 | ,604 |

## Direct Effects (Group number 1 - Default model)

|  | **SI** | **EE** | **PE** | **FC** | **BI** |
| --- | --- | --- | --- | --- | --- |
| **BI** | -,083 | ,261 | ,461 | ,000 | ,000 |
| **UTAUT4_FC02** | ,000 | ,000 | ,000 | 1,815 | ,000 |
| **UTAUT4_FC01** | ,000 | ,000 | ,000 | 1,000 | ,000 |
| **UTAUT4_SI02** | 1,188 | ,000 | ,000 | ,000 | ,000 |
| **UTAUT4_SI01** | 1,000 | ,000 | ,000 | ,000 | ,000 |
| **UTAUT1_EE03** | ,000 | 1,048 | ,000 | ,000 | ,000 |
| **UTAUT1_EE02** | ,000 | 1,120 | ,000 | ,000 | ,000 |
| **UTAUT1_EE01** | ,000 | 1,000 | ,000 | ,000 | ,000 |
| **UTAUT1_PE03** | ,000 | ,000 | ,932 | ,000 | ,000 |
| **UTAUT1_PE02** | ,000 | ,000 | ,819 | ,000 | ,000 |
| **UTAUT1_PE01** | ,000 | ,000 | 1,000 | ,000 | ,000 |
| **UTAUT1_BI01** | ,000 | ,000 | ,000 | ,000 | 1,821 |
| **UTAUT1_BI02** | ,000 | ,000 | ,000 | ,000 | 1,694 |
| **UTAUT1_BI03** | ,000 | ,000 | ,000 | ,000 | 1,217 |
| **UTAUT1_BI04** | ,000 | ,000 | ,000 | ,000 | 1,000 |

## Standardized Direct Effects (Group number 1 - Default model)

|  | **SI** | **EE** | **PE** | **FC** | **BI** |
| --- | --- | --- | --- | --- | --- |
| **BI** | -,080 | ,358 | ,635 | ,000 | ,000 |
| **UTAUT4_FC02** | ,000 | ,000 | ,000 | ,704 | ,000 |
| **UTAUT4_FC01** | ,000 | ,000 | ,000 | ,450 | ,000 |
| **UTAUT4_SI02** | ,817 | ,000 | ,000 | ,000 | ,000 |
| **UTAUT4_SI01** | ,628 | ,000 | ,000 | ,000 | ,000 |
| **UTAUT1_EE03** | ,000 | ,870 | ,000 | ,000 | ,000 |
| **UTAUT1_EE02** | ,000 | ,858 | ,000 | ,000 | ,000 |
| **UTAUT1_EE01** | ,000 | ,854 | ,000 | ,000 | ,000 |
| **UTAUT1_PE03** | ,000 | ,000 | ,858 | ,000 | ,000 |
| **UTAUT1_PE02** | ,000 | ,000 | ,816 | ,000 | ,000 |
| **UTAUT1_PE01** | ,000 | ,000 | ,859 | ,000 | ,000 |
| **UTAUT1_BI01** | ,000 | ,000 | ,000 | ,000 | ,917 |
| **UTAUT1_BI02** | ,000 | ,000 | ,000 | ,000 | ,906 |
| **UTAUT1_BI03** | ,000 | ,000 | ,000 | ,000 | ,706 |
| **UTAUT1_BI04** | ,000 | ,000 | ,000 | ,000 | ,604 |

## Indirect Effects (Group number 1 - Default model)

|  | **SI** | **EE** | **PE** | **FC** | **BI** |
| --- | --- | --- | --- | --- | --- |
| **BI** | ,000 | ,000 | ,000 | ,000 | ,000 |
| **UTAUT4_FC02** | ,000 | ,000 | ,000 | ,000 | ,000 |
| **UTAUT4_FC01** | ,000 | ,000 | ,000 | ,000 | ,000 |
| **UTAUT4_SI02** | ,000 | ,000 | ,000 | ,000 | ,000 |
| **UTAUT4_SI01** | ,000 | ,000 | ,000 | ,000 | ,000 |
| **UTAUT1_EE03** | ,000 | ,000 | ,000 | ,000 | ,000 |
| **UTAUT1_EE02** | ,000 | ,000 | ,000 | ,000 | ,000 |
| **UTAUT1_EE01** | ,000 | ,000 | ,000 | ,000 | ,000 |
| **UTAUT1_PE03** | ,000 | ,000 | ,000 | ,000 | ,000 |
| **UTAUT1_PE02** | ,000 | ,000 | ,000 | ,000 | ,000 |
| **UTAUT1_PE01** | ,000 | ,000 | ,000 | ,000 | ,000 |
| **UTAUT1_BI01** | -,150 | ,476 | ,839 | ,000 | ,000 |
| **UTAUT1_BI02** | -,140 | ,443 | ,780 | ,000 | ,000 |
| **UTAUT1_BI03** | -,100 | ,318 | ,560 | ,000 | ,000 |
| **UTAUT1_BI04** | -,083 | ,261 | ,461 | ,000 | ,000 |

## Standardized Indirect Effects (Group number 1 - Default model)

|  | **SI** | **EE** | **PE** | **FC** | **BI** |
| --- | --- | --- | --- | --- | --- |
| **BI** | ,000 | ,000 | ,000 | ,000 | ,000 |
| **UTAUT4_FC02** | ,000 | ,000 | ,000 | ,000 | ,000 |
| **UTAUT4_FC01** | ,000 | ,000 | ,000 | ,000 | ,000 |
| **UTAUT4_SI02** | ,000 | ,000 | ,000 | ,000 | ,000 |
| **UTAUT4_SI01** | ,000 | ,000 | ,000 | ,000 | ,000 |
| **UTAUT1_EE03** | ,000 | ,000 | ,000 | ,000 | ,000 |
| **UTAUT1_EE02** | ,000 | ,000 | ,000 | ,000 | ,000 |
| **UTAUT1_EE01** | ,000 | ,000 | ,000 | ,000 | ,000 |
| **UTAUT1_PE03** | ,000 | ,000 | ,000 | ,000 | ,000 |
| **UTAUT1_PE02** | ,000 | ,000 | ,000 | ,000 | ,000 |
| **UTAUT1_PE01** | ,000 | ,000 | ,000 | ,000 | ,000 |
| **UTAUT1_BI01** | -,074 | ,328 | ,582 | ,000 | ,000 |
| **UTAUT1_BI02** | -,073 | ,324 | ,575 | ,000 | ,000 |
| **UTAUT1_BI03** | -,057 | ,253 | ,448 | ,000 | ,000 |
| **UTAUT1_BI04** | -,048 | ,216 | ,383 | ,000 | ,000 |

## Minimization History (Default model)

| **Iteration** |  | **Negative eigenvalues** | **Condition #** | **Smallest eigenvalue** | **Diameter** | **F** | **NTries** | **Ratio** |
| --- | --- | --- | --- | --- | --- | --- | --- | --- |
| **0** | e | 12 |  | -,607 | 9999,000 | 1113,421 | 0 | 9999,000 |
| **1** | e | 12 |  | -,331 | 3,225 | 604,343 | 20 | ,355 |
| **2** | e* | 3 |  | -,258 | ,964 | 322,608 | 5 | ,894 |
| **3** | e* | 0 | 1444,188 |  | ,898 | 192,099 | 5 | ,765 |
| **4** | e | 1 |  | -,104 | ,599 | 166,512 | 4 | ,000 |
| **5** | e | 0 | 2305,356 |  | ,606 | 132,799 | 5 | ,690 |
| **6** | e | 0 | 662,678 |  | ,346 | 126,542 | 4 | ,000 |
| **7** | e | 0 | 846,513 |  | ,474 | 117,893 | 1 | 1,105 |
| **8** | e | 0 | 1017,968 |  | ,197 | 116,917 | 1 | 1,097 |
| **9** | e | 0 | 991,881 |  | ,056 | 116,867 | 1 | 1,052 |
| **10** | e | 0 | 1027,368 |  | ,006 | 116,866 | 1 | 1,007 |
| **11** | e | 0 | 1010,990 |  | ,000 | 116,866 | 1 | 1,001 |

## Model Fit Summary

## CMIN

| **Model** | **NPAR** | **CMIN** | **DF** | **P** | **CMIN/DF** |
| --- | --- | --- | --- | --- | --- |
| **Default model** | 51 | 116,866 | 68 | ,000 | 1,719 |
| **Saturated model** | 119 | ,000 | 0 |  |  |
| **Independence model** | 28 | 1071,468 | 91 | ,000 | 11,774 |

## Baseline Comparisons

| **Model** | **NFI Delta1** | **RFI rho1** | **IFI Delta2** | **TLI rho2** | **CFI** |
| --- | --- | --- | --- | --- | --- |
| **Default model** | ,891 | ,854 | ,951 | ,933 | ,950 |
| **Saturated model** | 1,000 |  | 1,000 |  | 1,000 |
| **Independence model** | ,000 | ,000 | ,000 | ,000 | ,000 |

## Parsimony-Adjusted Measures

| **Model** | **PRATIO** | **PNFI** | **PCFI** |
| --- | --- | --- | --- |
| **Default model** | ,747 | ,666 | ,710 |
| **Saturated model** | ,000 | ,000 | ,000 |
| **Independence model** | 1,000 | ,000 | ,000 |

## NCP

| **Model** | **NCP** | **LO 90** | **HI 90** |
| --- | --- | --- | --- |
| **Default model** | 48,866 | 22,785 | 82,817 |
| **Saturated model** | ,000 | ,000 | ,000 |
| **Independence model** | 980,468 | 878,772 | 1089,593 |

## FMIN

| **Model** | **FMIN** | **F0** | **LO 90** | **HI 90** |
| --- | --- | --- | --- | --- |
| **Default model** | 1,016 | ,425 | ,198 | ,720 |
| **Saturated model** | ,000 | ,000 | ,000 | ,000 |
| **Independence model** | 9,317 | 8,526 | 7,641 | 9,475 |

## RMSEA

| **Model** | **RMSEA** | **LO 90** | **HI 90** | **PCLOSE** |
| --- | --- | --- | --- | --- |
| **Default model** | ,079 | ,054 | ,103 | ,031 |
| **Independence model** | ,306 | ,290 | ,323 | ,000 |

## AIC

| **Model** | **AIC** | **BCC** | **BIC** | **CAIC** |
| --- | --- | --- | --- | --- |
| **Default model** | 218,866 | 234,166 |  |  |
| **Saturated model** | 238,000 | 273,700 |  |  |
| **Independence model** | 1127,468 | 1135,868 |  |  |

## ECVI

| **Model** | **ECVI** | **LO 90** | **HI 90** | **MECVI** |
| --- | --- | --- | --- | --- |
| **Default model** | 1,903 | 1,676 | 2,198 | 2,036 |
| **Saturated model** | 2,070 | 2,070 | 2,070 | 2,380 |
| **Independence model** | 9,804 | 8,920 | 10,753 | 9,877 |

## HOELTER

| **Model** | **HOELTER ,05** | **HOELTER ,01** |
| --- | --- | --- |
| **Default model** | 87 | 97 |
| **Independence model** | 13 | 14 |

## Execution time summary

| **Minimization:** | ,027 |
| --- | --- |
| **Miscellaneous:** | ,274 |
| **Bootstrap:** | ,000 |
| **Total:** | ,301 |

**Supplement 5**

Table S4 Results of the ANCOVA with acceptance as dependent variable and group (IG vs. CG) as independent variable while controlling for education and gender as covariates

| Source | Type III Sum of squares | df | Mean of squares | F | p | partial η2 |
| --- | --- | --- | --- | --- | --- | --- |
| Corrected model | 8,558a | 3 | 2,853 | 3,091 | ,030 | ,077 |
| constant term | 211,041 | 1 | 211,041 | 228,676 | <,001 | ,673 |
| Education high vs. low | 1,000E-5 | 1 | 1,000E-5 | ,000 | ,997 | ,000 |
| gender | 3,224 | 1 | 3,224 | 3,493 | ,064 | ,031 |
| IG vs. CG | 6,364 | 1 | 6,364 | 6,896 | ,010 | ,058 |
| Error | 102,440 | 111 | ,923 |  |  |  |
| overall | 1143,000 | 115 |  |  |  |  |
| Corrected total variation | 110,998 | 114 |  |  |  |  |
| R^2^ = ,077 (corrected R^2^ = ,052) | | | | | | |
